# Can the use of magnetized water affect the seedling development and the metabolite profiles of two different species: Lentil and durum wheat?

**DOI:** 10.3389/fpls.2022.1066088

**Published:** 2023-02-14

**Authors:** Sara Sestili, Cristiano Platani, Daniela Palma, Maria Assunta Dattoli, Romina Beleggia

**Affiliations:** ^1^ Council for Agricultural Research and Economics (CREA) Research Centre for Vegetable and Ornamental Crops, Monsampolo del Tronto, AP, Italy; ^2^ Council for Agricultural Research and Economics (CREA) Research Centre for Cereals and Industrial Crops, Foggia FG, Italy

**Keywords:** magnetic device, plant growth, plant metabolites, sustainability, water use efficiency

## Abstract

Seedlings of durum wheat and lentil were utilized to investigate the efficiency of magnetic water on growth and metabolic epicotyl profile. Tap water was passed through a magnetic device with a flow rate of max. 12900 - 13200 Gauss (G). Seeds and plantlets were grown on sand-free paper soaked by magnetized water, with unmagnetized tap water used in a control group. The growth parameters were collected at three time points (48, 96, and 144 hours after treatment), the same times at which metabolomic analysis was conducted on seeds, roots, and epicotyls. Although the effects varied with the species, tissues, and time point considered, compared with tap water (TW), the use of magnetized water treatment (MWT) led to higher root elongation in both genotypes. On the contrary, epicotyl length was not affected by treatment both in durum wheat and lentil. The results indicate that the use of magnetized water in agriculture can be considered a sustainable technology to promote plant development and quality with reduced and more efficient water usage, leading to cost-saving and environmental protection.

## 1 Introduction

Based on the changing climatic conditions and the rapidly growing world population estimated to reach 9 billion by 2050, agro-food production systems face the challenge of needing to rapidly increase food production by at least 70% (Lo Piccolo and Landi, 2021; Ye et al., 2022). Furthermore, food security is becoming an alarming problem across the globe. To achieve the goals of higher yields and healthier food, the application of novel technologies such as the use of magnetized water treatment (MWT) represent a valuable approach to contribute to the sustainability of water resources (Alderfasi et al., 2016; Zúñiga et al., 2016; Esmaeilnezhad et al., 2017). The use of irrigation MWT is known to reduce the effects of salt concentration, increase yield, quality, and plant tolerance to biotic and abiotic stresses, and improve plant water use efficiency (WUE) in some crops (Maheshwari and Grewal, 2009; Hozayn et al., 2011; Hozayn et al., 2013; Hachicha et al., 2018; Akrimi et al., 2021). The first commercial magnetic device for water treatment was realized in 1958 by Vemeiren (1958), and current devices are environmentally friendly, competitively priced, and have no energy requirements (Ben Amor et al., 2017). The water is magnetized as it passes through a strong magnetic field (MF) that acts through two mechanisms: on the ions and molecules in the solution (ion mechanism), and on nuclei and particles already present in the solution (a surface mechanism) (Chibowski and Szcześ, 2018). The so-called “magnetic water” has been widely studied and adopted in industry, medicine, and agriculture (Surendran et al., 2016; Chibowski and Szcześ, 2018). MWT has a beneficial effect on common human conditions, such as atherosclerosis, arthritis pain, kidney and gall bladder stones, circulatory system normalization, aging, fatigue prevention, and weight control (Lee and Kang, 2013; Moosa et al., 2015; Raafat and Nabil, 2016; Ebrahim and Azab, 2017; Goyal et al., 2017). MWT also has beneficial effects on animals, helping them to live longer and healthier lives, with increased production (milk, wool, meat, eggs), growth performance, nutrient digestibility, and optimization of the rumen fermentation parameters (Gholizadeh et al., 2008; Sargolzehi et al., 2009; Ebrahim and Azab, 2017; El-Hanoun et al., 2017). The use of magnetically treated water in agriculture could be considered a sustainable method since it saves water consumption, and mitigates the harmful effects of drugs, toxins, and environmental pollutants by reducing methane production, which contributes to mitigating the environmental impact on livestock (Tyari et al., 2014; Sharma et al., 2018). The effects of a static magnetic field on seed germination, seedling development, and plant yield have been reported (Maffei, 2014; Chadapust et al., 2017; Nyakane et al., 2018; Abdollahi et al., 2019; Rifna et al., 2019). Factors such as polarity, intensity, exposure time, device magnet type, and the genotype-dependent effect can influence plant development positively or negatively (Surendran et al., 2016). On the contrary, irrigation MWT has resulted in a higher crop yield and soil properties even with low-quality water (Mohamed and Ebead, 2012; Ali et al., 2014; El-Kholy et al., 2015). The magnetic field influences the hydrogen bond-related properties of water in surface tension, evaporation rate, conductivity, salts solubility, pH, and molecular clustering of water, making it softer, lighter, and more easily absorbable by plants (Ospina-Salazar et al., 2018; Al-Bahrani, 2018; Wang et al., 2018; Pang, 2014; Alattar et al., 2020). However, although the effects on plant development have been investigated for at least half a century, the understanding of magnetic field action on water is still unclear. Positive impact on growth parameters (seed germination, shoot and root growth, emergence rate), soil (essential element uptake, mobility of nutrients from fertilizers, soil electrical conductivity, water holding capacity, soil pH), and water properties (water viscosity, surface tension, vaporization rate, water pH) were reported (Maheshwari and Grewal, 2009; Maffei, 2014). The beneficial effects of irrigation MWT on plant growth characteristics depend upon the species, the pathway length in the magnetic field, and the flow rate (Teixeira da Silva and Dobránszki, 2014; Surendran et al., 2016; Hachicha et al., 2018). The effect on seed germination and plant yield is reported in several species and is related to an increase in green and dry biomass, mobility, and micronutrient uptake (Fe, Zn, Mn), and higher photosynthetic activity due to greater total chlorophyll content compared to those irrigated with untreated water (Moussa, 2011; El-Sayed, 2014; Yusuf and Ogunlela, 2015; Haq et al., 2016; Shahin et al., 2016; Abd-Elrahman and Shalaby, 2017; Ospina-Salazar et al., 2018; Hozany and Qados 2010; Samarah et al., 2021). A previous study reported a significant effect on wheat and tomato seedling growth (Sestili et al., 2017). However, the MWT effect on plant metabolome is not yet widely studied. Based on this, we compared the effects of MWT and tap water on growth parameters and metabolomic profile of the seeds, roots, and epicotyls of durum wheat and lentil seedlings. This study aims to shed light on the differences in the level of metabolites involved in early plant development under this still controversial and seldom accepted technology.

## 2 Material and methods

### 2.1 Plant material and experimental design

Seedlings of durum wheat and lentil were grown in MWT and tap water (TW). Seeds of durum wheat (*Triticum durum* Desf.) variety PR22D89 provided by CREA-CI and the commercial lentil (*Lens culinaris* Medik or *Lens esculenta*) variety “Colfiorito” were used. Seeds of both species were calibrated for size and weight (thousand seed weight) and grown on top of 15 cm x 11 cm germination paper and watered with 3.3 ml of treated or untreated water. The germination paper was inserted in a numbered plastic bag and randomly positioned on non-transparent rigid plastic panels. All panels were placed vertically in the growth chamber under 20°C temperature, 50% relative humidity, for a 16 h photoperiod (50 µmol m-2s-1) provided by cool white fluorescent lamps (Fluora L30W/77 Osram) (**Supplementary Figure 1**). Material was tested and collected from both species at designated time points: control (t0), 48 (t1), 96 (t2), and 144 (t3) hours after sowing. Seven independent trials for each species (from three to two for each time point) were planned by scaling the sowing throughout the three time points to register the growth parameters and collect enough vegetable material for metabolomic analysis. A total of seven experiments were planned for both species. The plant numbers differed for species and time points. For wheat, we tested 300 plants at t1 and 200 plants at t2 and t3. For lentil, we tested 300 plants at both t1 and t2 and 100 plants at t3. After detecting the growth parameters, the vegetable material was frozen, lyophilized, and sent to CREA-CI for metabolomic analysis.

### 2.2 Magnetic device

The OverWater magnetic device is a product of OVERTIS S.r.l (Forli, Italia). We used the model 110/140 (Ambient Noise Level: 0 d B, Inner magnetic field: max. 12900 - 13200 Gauss (G), Storage Temperature: - 20°C/+ 50°C). The device is a sort of “conveyor system” of natural magnetic flux that allows the device to act as a resonator of some substances that vary the flow of information of the water molecules passing through it. The device does not require any connection to the electric grid and does not house any type of accumulator. Therefore, it is free of electromagnetic emissions. The only magnetic field involved is the terrestrial one, combined with the static field generated by some neodymium magnets. The magnets are positioned and oriented to allow the correct functioning of the device. It works even when the orientation is not aligned with the terrestrial poles or in the case of installation occurring near the terrestrial equator, where the natural magnetic field assumes a value equal to about 1/3 of the one present near the poles.

### 2.3 Water treatment and analysis

Tap water was passed through the OverWater magnetic device. The conditions of the tap water withdrawal were the same for each experiment but slightly different between the two species. Water pH and electric conductivity (EC) were detected, at the beginning of each experiment, before and after passing through the magnetic device. The dry residue was measured in 500 ml of water at 180°C.

### 2.4 Growth parameters and metabolomic analysis

For both species, total root length (TRL) was manually measured at all time points relative to the primary and lateral roots. Epicotyl length (EL) was measured starting from the root base to the tip. The data were collected at t1, t2, and t3 for roots and only at t2 and t3 for epicotyls. Seeds, roots, and epicotyls collected at all time points were used for metabolomic analysis in both species and treatments. At the t0 time point, the metabolomic profile of seeds was detected. Five and six repetitions were used for wheat and lentil, respectively. The freeze-dried samples, milled with an agate jar and balls (Pulverisette 7 Planetary Micro Mill, Classic Line; Fritsch GmbH Milling and Sizing, Idar-Oberstein, Germany), were stored at −20°C until analysis. Sample extraction, derivatization, and the analysis of the metabolites by gas chromatographymass spectrometry were performed as reported by Beleggia et al. (2013) with few modifications. Briefly, for each sample, 100 mg of seeds and 30 mg of roots and epicotyls were extracted using a mixture of methanol, water, and chloroform (1:1:3 v/v/v), and aliquots of the polar (50 μL) and non-polar phase (500 μL) were dried (Speedvac Jouan RC1022, Thermo Electron Corporation, USA). The polar fraction was redissolved and derivatized for 90 min at 37°C in methoxyamine hydrochloride in pyridine (70 μL; 20 μg/mL), followed by incubation with N-methyl-N-(trimethylsilyl) trifluoroacetamide (MSTFA, 120 μL) at 37°C for 30 min. The non-polar fraction was redissolved and derivatized for 30 min at 37°C in MSTFA (70 μL). The polar and non-polar metabolites were redissolved, derivatized, and analyzed using gas chromatography (6890N; Agilent Technologies, Santa Clara, CA, USA) coupled to a quadrupole mass spectrometer (7000B, Agilent Technologies). For both fractions, the injection volume was set at 1μL, and the metabolites were separated on an HP-5ms capillary column (60 m, 0.25mm i.d., 0.25 μm film thickness) using Helium as carrier gas at the flow of 1mL/min. The injection temperature, transfer lines, and ion source were set at 280°C and 250°C for polar and non-polar metabolites, respectively. For polar fraction, the oven was kept at 70°C for 1 min, increased by 5°C/min up to 310°C, held for 15 min, then increased to 340°C for 1 min; the scan range was from 30 to 700 amu, and the mass spectra were recorded at 2.21 scan/s. For the non-polar fraction, the oven was kept at 70°C for 5 min, increased by 5°C/min up to 310°C, and held for 1 min; the scan range was from 50 to 700 amu and the mass spectra were recorded at 2.28 scan/s. The MassHunter Qualitative Analysis program was used to evaluate all chromatograms and mass spectra. The metabolites were identified by comparing the mass spectral data with those of a custom library obtained with reference compounds and the NIST 2011 database. The metabolites were quantified using the MassHunter Quantitative Analysis program using the corresponding calibration curves. The standards and all chemicals used were HPLC grade (Sigma Aldrich Chemical Co., Deisenhofen, Germany).

### 2.5 Statistical analysis

The effects of MWT versus TW on seed, root, and epicotyl development, and the interaction with timing, were analyzed using two-way ANOVA (analysis of variance) by using five and six biological replicates for durum wheat and lentil, respectively. Means of growth data were compared using the “Standard Least Squares” method and were separated by Student’s unpaired t-test α=0.05 p ≤ 0.05. The metabolites were statistically analyzed, following a randomized model taking into account the amount of metabolite in the tissues tested over T treatments and t timing. The *post hoc* Tukey’s test, at the significance level of α=0.05 (p ≤ 0.05), was used to compare the means of each metabolite during timing. JMP v. 8 (SAS Institute Inc.) was used to analyze morphological and metabolomic data. The metabolites significant for T and t×T interaction were subjected to principal component analysis (PCA) based on correlations, using PAST v. 3.15 (Hammer et al., 2001). Hierarchical cluster analysis (HCA), based on Ward’s method with the two-way cluster option, was used to group the metabolites based on treatment and tissue to obtain a general comprehensive characterization of samples of both species (JMP v. 8).

## 3 Results

Water properties before and after magnetization for both species are reported in **Table 1**. The pH slightly decreased after the treatment in both species and timing except for lentil at t144. On the contrary, the levels of dry residue were higher in the treated water in durum wheat and lentil.

**Table 1:** Water properties before and after magnetic treatment.

Species	parameters	48		96		144	
		TW	MWT	TW	MWT	TW	MWT
**Drurum Wheat**	pH	7.42	7.28	7.34	7.25	7.52	7.20
	dry residue mg/l	1098	1111	1100	1145	1101	1143
	flow velocity m/s	1.76	1.76	1.65	1.65	1.80	1.80
**Lentil**	pH	7.68	7.51	7.66	7.51	7.68	7.52
	dry residue mg/l	1115	1150	1100	1139	1090	1108
	flow velocity m/s	1.93	1.93	1.97	1.97	1.86	1.86

### 3.1 Growth measurements

Total root length (TRL) and epicotyl length (EL) were recorded at the three time points in durum wheat and lentil (**Table 2**). Significantly, seedlings grown on paper treated with magnetized water recorded higher root and epicotyl length values compared with those developed on paper with untreated water (**Table 2**). Among all growth parameters, the TRL derived by measuring the primary and secondary (left and right) roots was statistically significant after treatment at t3 for wheat and t1 for lentil (**Table 2**). The TRL of both species exhibited an increasing trend with treatment along the experimental time points. In wheat, the TRL ranged from 20.5 cm to 554.4 cm in MWT in contrast to 19.5 cm to 519.6 cm in TW. In lentil, TRL ranged from 25.6 cm to 69.8 cm in MWT and from 21.9 to 57.2 in TW. Although EL was longer on water-treated paper, it was not statistically significant in both species nor at each time point (**Table 2**). In both species, the effect of treatment and timing on the developing seedlings was investigated. As expected, a significant influence due to timing was observed in both species and for all growth parameters. The TRL was positively affected by treatment both in durum wheat (F ratio 9.998, p> 0.0042) and lentil (F Ratio 7.3870, p<0.0108). The timing*treatment (t×T) interaction was not statistically significant for roots and epicotyl in both species (**Table 3**).

**Table 2:** ANOVA significance for growth parameters of durum wheat and lentil.

Time points	Durum wheat				Lentil			
	Total root		Epicotyl		Total root		Epicotyl	
	TW	MWT	TW	MWT	TW	MWT	TW	MWT
48	19.5 ± 2.3^a^	20.5 ± 2.9^a^	0.0	0.0	21.9 ± 0.5^b^	25.6 ± 0.5^a^	0.0	0.0
96	262.9 ± 7.3^a^	281.1 ± 10.1^a^	34.9 ± 1.0^a^	36.4 ± 1.6^a^	44.1 ± 1.0^a^	47.8 ± 2.8^a^	34.8 ± 1.7^a^	35.2 ± 1.4^a^
144	519.7 ± 5.9^b^	554.4 ± 9.3^a^	69.5 ± 2.7^a^	77.4 ± 3.4^a^	57.2 ± 4.3^a^	69.8 ± 5.2^a^	66.0 ± 2.6^a^	71.2 ± 2.2^a^

p = <,0001*. Different letters indicate significant differences at p<,0001.

**Table 3:** Statistical analysis of all growth parameters according to a two-way ANOVA for timing (t), Treatment (T), and their interaction (t x T) in both species.

Parameters	Durum wheat				Lentil			
	Total root		Epicotyl		Total root		Epicotyl	
	F Ratio	Prob > F	F Ratio	Prob > F	F Ratio	Prob > F	F Ratio	Prob > F
timing	2760,1528	<,0001*	261,2750	<,0001*	87,3825	<,0001*	276,1486	<,0001*
Treatment	9,9977	0,0042*	4,0308	0,0619	7,3870	0,0108*	1,9492	0,1780
timing*Treatment	2,9527	0,0714	1,8221	0,1959	1,5001	0,2394	1,3777	0,2543

The * meaning is significant at p<,0001.

### 3.2 Metabolomic analysis

#### 3.2.1 Metabolite characterization of durum wheat and lentil

The durum wheat and lentil grains used in the study were characterized for metabolite composition at the t0 time point (no treated water) before starting the experiment (**Supplementary Table 1**). 39 and 44 metabolites were detected in durum wheat and lentil grains, respectively. These metabolites belong to different classes: amino acids, organic acids, sugars and sugar alcohols, fatty acids, polycosanol, alkylresorcinols, tocopherols, and phytosterols. The two species have several metabolites in common, although in each one we observed the presence of specific metabolites of different compound classes: e.g., alkylresorcinols were detected only in durum, and tocopherols and phytosterol in lentil. In durum wheat, raffinose was the most common, followed by but to a lesser extent sucrose, turanose and phosphate; among apolar metabolites, polyunsaturated fatty acids (PUFA) were present in higher amounts (linolenic acid > linoleic acid > oleic acid). In lentil, the most abundant metabolites were sucrose and maltitol for polar compounds, and hexadecenoic acid and campesterol for apolar metabolites.

#### 3.2.2 Metabolite profile of durum wheat

**Figure 1:**
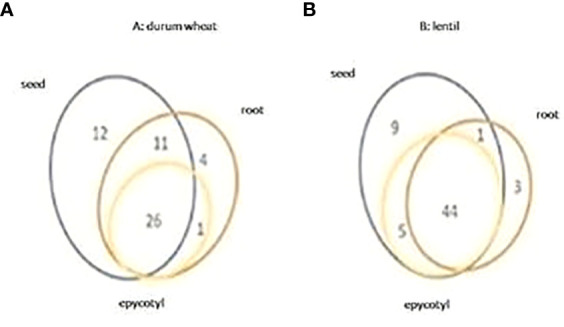
Venn diagram of the metabolites detected in all tissues analyzed in durum wheat **(A)** and in lentil **(B)**.

A total of 58 metabolites were identified and quantified using GC-MS during the course of the experiment. The data were analyzed using two-way ANOVA with significant differences valued at p ≤ 0.001. Among all metabolites, 26 were common to all tissues, and 11 common to seeds and roots. Only sitosterol was detected in both roots and epicotyls, and 12 and 4 metabolites were detected only in seeds and roots, respectively (**Figure 1A**). All metabolites significantly differed with timing, except oxalic acid in roots, and mannose, mannitol, sorbitol, sucrose, and linolenic acid in epicotyl (**Supplementary Table 2**). In seeds, the treatment strongly affected oxalic acid, fumaric acid, docosane, and 1,3-dihydroxy-5-eicosylbenzene. In addition to these metabolites, four sugars (sucrose, maltose, turanose, raffinose), one fatty acid (decanoic acid), and one alkylresorcinol compound (1,3-dihydroxy-5-nonadecylbenzene) were only significantly affected by the t×T interaction (**Supplementary Table 2**). In roots, the treatment had significant effects on 19 sugar compounds, among which 11 were significantly affected also by the t×T interaction (**Supplementary Table 2**). In epicotyls, the treatment significantly affected 6 sugars, which, with the exception of fructose, were also influenced by the t×T interaction (**Supplementary Table 2**). In addition, 7 further sugars were significantly influenced only by the t×T interaction. **Supplementary Figure 2** reports the percentage of the compounds affected by the timing, treatment, and their interaction in seed, root, and epicotyl. Considering the metabolites common to all tissue analyzed, sucrose and turanose were the most significantly affected by the t×T interaction (**Supplementary Table 2**).

#### 3.2.3 Metabolite profile of lentil

In lentil, a total of 62 metabolites were identified. Among these: 44 were common to all tissues, 9 were detected only in seeds, and 3 only in roots; 1 was shared among seeds and roots, and 5 were detected in seeds and epicotyls (**Figure 1B**). As observed for durum wheat, almost all the metabolites detected in lentil were significantly modulated by timing, while different behavior was showed for the treatment over the tissues analyzed. In particular, 13 (mostly sugars), 13 (mostly amino acids), and 7 (amino acids and sugars) metabolites were significantly affected in seeds, roots, and epicotyls, respectively. Among the 13 seed metabolites modulated by the treatment, seven were also significant for the t×T interaction in addition to threonine, sorbitol, and campesterol (**Supplementary Table 3**). In roots, all the metabolites affected by the treatment were also significant for the t×T interaction except for serine, phosphate, and myristic acid. Aconitic acid and galactose were significantly affected only by the timing and treatment interaction. In epicotyls, only mannitol and maltitol were significantly affected by the t×T interaction (**Supplementary Table 3**). Significantly higher percentages of metabolites were detected in lentil roots due to treatment and t×T interaction (**Supplementary Figure 2**).

#### 3.2.4 Multivariate analysis of dataset

**Figure 2:**
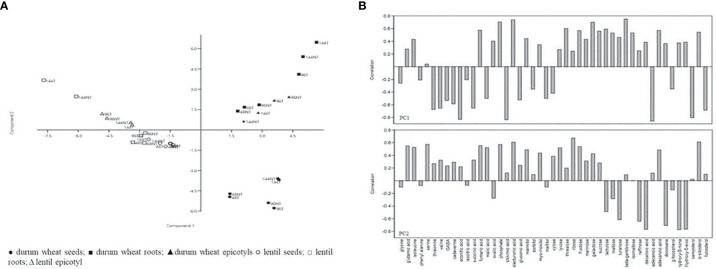
PCA analysis of combined dataset of the two species. **(A)** the score plot of the first two PCs; **(B)** correlation of metabolites to PC1 and PC2.

**Figure 3:**
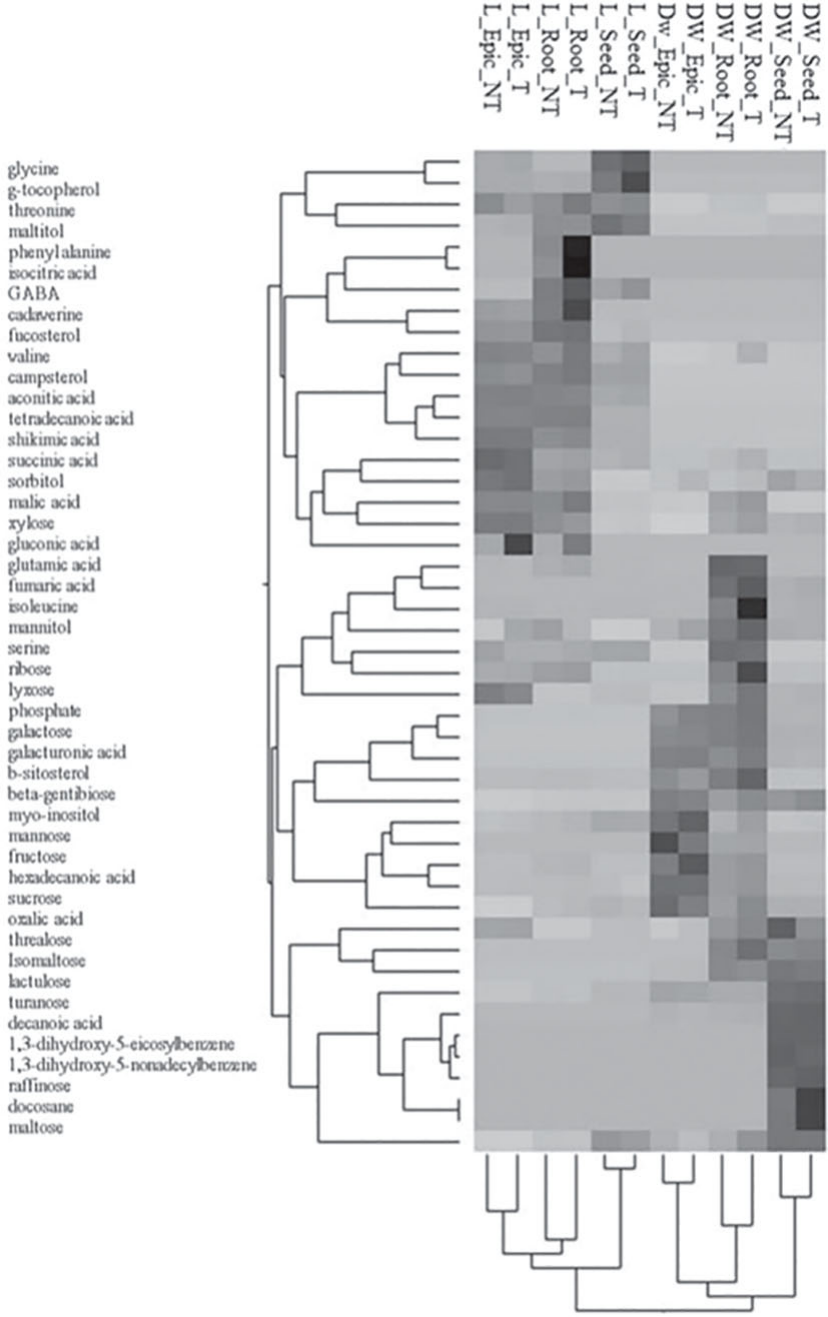
Two-way hierarchical clustering (Ward’s method) of metabolites data for durum wheat and lentil.

The metabolites significant for T and t×T interaction in both species were used in combination for the PCA analysis to evaluate the relationship among metabolites, growth, and treatments. The first two principal components explained 47.4% of the total variance estimated: PC1 explained 28.5%, while PC2 explained 18.9% and were correlated, positively and negatively, to the metabolites according to **Figure 2B**. The score plot of the first two PCs (**Figure 2A**) identified three well-defined clusters. PC1 separated the species, whereas PC2 differentiated the tissue samples of durum wheat (**Figure 2A**). Within the species, timing well discriminates roots and epicotyls at 96h, while the differentiation due to treatment increased with time and is more evident in the roots of both species, reflecting the different content of metabolites represented by the two components (**Figure 2A**). **Figure 3** shows the results of the hierarchical clustering analysis (HCA) based on the Ward method using the average data of metabolites significant for T and t×T of the two species. The obtained dendrogram distinguishes the samples into two main groups related to the species. Each group is divided into different subgroups based on tissues and treatment. The first subgroup includes treated and non-treated seeds, while the second subgroup comprises two clades for treated and non-treated root and epicotyl. The metabolites are clustered in three main groups based on the cluster of the two species. The first group comprises four subgroups, which include almost all the metabolites significant for T and t×T and are mainly present in almost all lentil tissues. The second group, divided into three subgroups, contains mainly the significant root and epicotyl metabolites of durum wheat except for serine, galactose, and hexadecanoic acid which is significant only in lentil. The third cluster, divided into two clades, includes the metabolites significant only in durum wheat except for maltose which is significant in both species. **Supplementary Tables 4, 5** highlight that some metabolites are characteristic of one or the other species. In durum wheat, some metabolites were significantly detected following treatment (fumaric acid, galacturonic acid, turanose, isomaltose, raffinose, decanoic acid, docosane, 1,3-5-nonadecylbenzene, and 1,3-5-eicosylbenzene) while those significant only in lentil were glycine, phenylalanine, GABA, cadaverine, aconitic acid, isocitric acid, gluconic acid, tetradecanoic acid, g-tocopherol, campesterol, and fucosterol. In both species, most of the significant metabolites were detected in higher amounts following the treatment in each tissue. In durum wheat, the metabolites highly abundant after treatment were turanose, sucrose, and lactulose in seeds, and phosphate, sucrose, and fructose in roots and epicotyls. Furthermore, all the root metabolites increased with the treatment except isomaltose, whose content was higher in control than in treated samples. In lentil, aconitic acid, glycine, and phosphate were highly abundant in treated seeds, while sucrose and malic acid amounts increased after treatment, both in root and epicotyl.

#### 3.2.5 Relationship between morphological traits and metabolites

To evaluate the possible relationship between morphological traits and metabolite content due to the treatment, bivariate analysis was performed for each species and tissue by considering only the metabolites significantly different for T and t×T interaction. **Table 4** reports the correlation between metabolites and total root length, and between metabolites and epicotyl length of durum wheat samples not treated (NT) and treated (T) with magnetized water. In the root samples, 20 and 17 significant correlations were identified in NT and T samples, respectively. All the metabolites were correlated positively except sucrose, which correlated negatively to the root development. The metabolite valine, myo-inositol, and lactulose lost correlational significance with treatment. The amount of these metabolites was higher in treated roots (**Supplementary Table 4**). For the durum wheat epicotyl, the metabolites correlated in the NT and T samples were ten and nine, respectively. Phosphate, shikimic acid, myo-inositol, and lactulose were positively correlated only in the NT samples, while oxalic acid, mannitol, and sucrose were positively correlated with epicotyl length exclusively in the T sample group. Higher amounts of all these metabolites were found in the treated samples, except for sucrose, more of which was detected in non-treated epicotyl (**Supplementary Table 4**). In general, higher variability was observed in the epicotyl among the correlations’ versus for NT and T samples e.g., mannose was strongly correlated in both groups but negatively and positively for NT and T, respectively. Mannose was more representative in epicotyl than the other tissues and the non-treated samples compared to treated ones (**Supplementary Table 4**). As for durum wheat, **Table 5** reports the bivariate results for root and epicotyl of lentil in NT and T samples. Thirteen metabolites correlated significantly with the root length in both groups; only galactose and tetradecanoic acids correlated significantly in NT and T, respectively. Although these metabolites were scarcely detected in all lentil tissues, the treated roots showed a higher amount of both (**Supplementary Table 5**). Concerning the lentil epicotyl of NT samples, only threonine and myo-inositol showed significant positive and negative correlations, respectively. Differently, correlations with glutamic acid, mannitol, maltitol, and ribose also became significant in treated samples. There were higher amounts of all these metabolites in the treaed samples, except for glutamic acid (**Supplementary Table 5**).

**Table 4:** Correlation between morphological traits (root and epicotyl lengths) and metabolites’ content for non-treatment (NT) and treatment (T) conditions in durum wheat.

	NT			T		
	Rsquare	F ratio	Prob>F	Rsquare	F ratio	Prob>F
root						
valine	0.67	8.2691	*	0.18	2.8726	n.s.
isoleucine	0.39	26.4761	***	0.71	31.9858	****
succinic acid	0.61	20.4618	***	0.45	10.6962	**
malic acid	0.98	614.7543	****	0.79	47.8979	****
phosphate	0.66	25.8551	***	0.37	7.5685	*
shikimic acid	0.80	52.4073	****	0.74	38.0344	****
galacturonic acid	0.79	47.6200	****	0.74	36.3883	****
sorbitol	0.83	61.7990	****	0.72	32.7788	****
myo-inositol	0.67	26.2313	***	0.19	3.1679	n.s.
xylose	0.90	115.4550	****	0.55	16.0387	**
lyxose	0.89	106.0024	****	0.82	58.2758	****
threalose	0.87	87.6004	****	0.80	52.2118	****
ribose	0.89	102.6313	****	0.76	42.5458	****
fructose	0.84	67.1065	****	0.57	17.4025	**
sucrose	0.38	8.1061	*	0.34	6.6438	*
lactulose	0.89	109.5432	****	0.25	4.3550	n.s.
maltose	0.89	101.6755	****	0.90	119.2440	****
turanose	0.77	44.5621	****	0.76	40.6815	****
b-gentibiose	0.79	50.1846	****	0.29	5.2579	*
isomaltose	0.78	47.2196	****	0.84	70.8313	****
epycotil						
oxalic acid	–	–	–	0.60	11.8593	**
phosphate	0.88	57.5467	****	0.39	5.0314	n.s.
shikimic acid	0.53	9.1027	*	0.02	0.1648	n.s.
galacturonic acid	0.77	26.7502	***	0.48	7.4555	*
mannitol	0.33	3.9115	n.s.	0.50	8.0839	*
myo-inositol	0.74	23.1477	**	0.16	1.5909	n.s.
fructose	0.82	35.6245	***	0.63	13.5579	**
mannose	0.80	32.1288	***	0.86	47.2555	****
sucrose	0.11	1.0309	n.s.	0.42	5.7203	*
lactulose	0.59	11.7821	**	0.14	1.2797	n.s.
turanose	0.72	20.9784	**	0.69	18.0517	**
isomaltose	0.94	128.2777	****	0.95	147.2269	****
b-sitosterol	0.92	91.9815	****	0.72	20.5367	**

* P< 0.05; ** p< 0.01; *** p< 0.001; **** p< 0.0001; n.s., not significant.

**Table 5:** Correlation between morphological traits (root and epicotyl lengths) and metabolites’ content for non-treatment (NT) and treatment (T) conditions in lentil.

	NT			T		
	Rsquare	F ratio	Prob>F	Rsquare	F ratio	Prob>F
root						
glutamic acid	0.59	23.3136	***	0.58	21.8476	***
phenyl-alanine	0.57	21.5170	***	0.59	22.9594	***
serine	0.48	14.9386	**	0.55	19.3472	***
threonine	0.46	13.4167	**	0.60	24.0136	***
valine	0.54	18.9432	***	0.68	33.4161	****
GABA	0.56	20.2050	***	0.58	22.5488	***
cadaverine	0.47	14.4070	**	0.56	20.2655	***
aconitic acid	0.84	85.9923	****	0.81	66.5183	****
isocitric acid	0.53	18.542	***	0.53	17.9766	***
malic acid	0.41	11.0871	**	0.49	15.5868	**
phosphate	0.64	28.2451	****	0.80	64.5236	****
gluconic acid	0.51	16.8544	***	0.64	28.9313	****
galactose	0.58	21.7833	***	0.02	0.3201	n.s.
tetradecanoic acid	0.18	3.6104	n.s.	0.61	25.6549	****
g-tocopherol	0.74	46.8317	****	0.63	27.0844	****
epycotil						
glutamic acid	0.01	0.1029	n.s.	0.46	8.4203	*
threonine	0.36	5.6115	*	0.45	8.1870	*
mannitol	0.05	0.5158	n.s.	0.90	91.6580	****
myo-inositol	0.88	73.3935	****	0.88	72.3606	****
maltitol	0.07	0.7920	n.s.	0.74	28.6942	***
ribose	0.16	1.9234	n.s.	0.66	19.1955	**

* P< 0.05; ** p< 0.01; *** p< 0.001; **** p< 0.0001; n.s., not significant.

## 4 Discussion

One of the priorities for agricultural research is to address the variations in plant growth environments caused by climatic changes that create adverse conditions that destabilize agricultural systems and endanger world food production (Anderson et al., 2020). Many studies have been conducted at species, organ, and tissue levels to understand the mechanisms of resistance, and the molecular and physiological responses that lead to greater tolerance to various abiotic stresses to preserve yield and quality of productions (Nakabayashi and Saito, 2015; Haak et al., 2017; Vives-Peris et al., 2020; Zhang et al., 2022). However, the evaluation of the possible use of novel technologies is still in progress (Okasha et al., 2022). In this regard, studies on the effect of magnetic fields (MF) on economically important plants have increased in the last decades. Many researchers reported the positive effect of MF on plant production and development, fruit quality, biotic stress resistance, water and nutrient uptake, and consequently higher WUE and NUE. On the contrary, the effects of using irrigation water treated with a magnetic field are poorly studied. In this work, the growth parameters and metabolite profile of durum wheat and lentil seedlings treated with magnetized water were investigated. The results showed that MWT significantly increased the plantlets development in both species, in agreement with previous investigations in other species in which the use of magnetized irrigation water promotes seed germination and plant growth even with low-quality water (Teixeira da Silva and Dobránszki (2014); El-Sayed, 2014; Elhindi et al., 2020; Akrimi et al., 2021; Liu et al., 2022; Okasha et al., 2022 and references therein). Magnetized treated water, also defined as “structured water”, has altered hydrogen bond structures that might directly or indirectly affect several physiological processes in plants leading to incremental growth and development (Nasher, 2008; Teixeira da Silva and Dobránszki 2014; Zúñiga et al., 2016; Lindinger, 2021). The pH of water slightly decreased with magnetization in both species during the treatment, in contrast with those reported by other authors who observed an increase in the pH value. This probably depends on the type of water used (tap, ground, lake, reservoir water) due to differences in mineral and organic material contents, and on the exposure to factors (light, heat, and mechanical disturbances) that could affect the magnitude, type, and stability of structuring (Chibowski and Szcześ, 2018; Lindinger, 2021). The lower pH of treated water is probably due to mainly fully protonated species in solution that can affect the mobility and availability of metal ions and thus the plant growth (Dai et al., 2014; Ponce et al., 2015). The treated water showed a higher dry residue with respect to the control tap water due to the higher frequency of collisions between ions on opposite sides, combining to form a mineral precipitate or insoluble compound (Gholizadeh et al., 2008). The MF water treatment led to a “memory effect” (or residual effect) that remains immediately after removing the MF and is based on the time and intensity of the treatment (Pang and Deng, 2008). For this reason, the influence of MWT continues after the treatment and along with plant development. The magnetization could regulate the uptake and translocation of mineral nutrients from the roots to the aboveground parts by affecting root morphology (Liu et al., 2022). Furthermore, in potatoes, tomatoes, and lentils, the MWT increased the diameter of aerial stem structure, indicating a possible cambium differentiation to xylem and phloem, and an improving photoassimilate translocation (Qados and Hozayn, 2010; Selim and El-Nady, 2011; Hozayn et al., 2016). In our study, the total root (principal and lateral roots) of both species treated with MWT showed a greater development than the control plants. Considering timings, the differences between treated and non-treated plants were more evident for lentil at the early stage (t1) but later in the growth of durum wheat (t3). This result is probably due to the differing root development and morphology of the two species; in durum wheat, the lateral roots were significantly influenced by MWT, while in lentil, only the principal root was affected by treatment (data not shown). The epicotyls of both species were not significant following treatment. In comparison to other works, our trials were conducted in a controlled environment and free-soil conditions to avoid any external influences. The use of plantlets has limited deeper investigation into the effect of MWT on plant development, since the last data were collected at a plant height of less than 100 cm. However, this also allowed us to highlight the metabolomic profile modifications that the plant undergoes at the early growth stage after treatment. In a previous study on wheat, durum wheat, and tomatoes grown on paper with sand treated with MW, we registered higher plant development (root and epicotyl) in wheat and tomatoes than in the controls (Sestili et al., 2017). Wheat and lentil were both used in previous studies on the influence of magnetized irrigation water on plant growth and under different stress conditions (Ćirković et al., 2017; Abd-Elrahman and Shalaby, 2017; Qados and Hozayn, 2010; Selim et al., 2019). In our work, we performed metabolomic analysis on seeds, roots, and epicotyls in both species at the same time points in which the growth parameters were collected to pinpoint a possible correlation between metabolome profile variation and plantlet development under the use of MWT. In our study, the metabolomic profile analysis of plants grown with MWT highlighted a significant increase for most of the metabolites in comparison to the use of tap water. Primary metabolites are directly involved in normal growth, development, and reproduction. Despite the increase in the concentration of the metabolites after using MWT, the correlation with root and epicotyl elongation was significantly different, highlighting that in both species, amino acids in roots and sugars in epicotyls are mainly positively affected by MWT, resulting in a higher or even “new” correlation with the growth parameters with respect to the control plantlets. The durum wheat epicotyl grown with MWT showed a positive correlation with oxalic acid and sugars with respect to controls. It is interesting to note that the oxalic acid content in durum wheat treated seeds was lower than the controls, indicating a positive effect of MWT since the consumption of foodstuffs rich in oxalic acid can induce serious health problems called hyperoxaluria that lead to kidney stones, bone disease, and anemia (Siener et al., 2006). The sugars positively correlated with epicotyl had different amounts; mannitol increased while sucrose decreased, indicating a dose dependent effect. El-Sayed reported that in broad bean treated with magnetic water, sugars increased because of the close relationship between stomatal conductance and photosynthesis (2014). Mannitol plays an important role in plant growth, photosynthesis, and abiotic stress tolerance by acting as an osmoprotectant (Claeys et al., 2014; Zulfiqar et al., 2020). Photosynthetic carbon converted to sucrose is vital for plant growth. Sucrose acts as a signaling molecule and a primary energy source that coordinates the source and sink development. The higher amount of some sugars was positively correlated with epicotyl, confirming the important role of mannitol in plant growth. In lentil, among the fatty acids, hexadecanoic acid and tetradecanoic acid were significantly influenced by MWT, but only tetradecanoic acid was positively correlated with root elongation. This confirms the role of saturated fatty acid in the biosynthesis and/or regulation of fundamental components for plant growth and seed development (Bonaventure et al., 2003). As far as we know, no other works have reported the effect of using MWT on plant metabolomic profiles. In other species, including wheat, a significant increase in photosynthetic pigment, total indole and phenol contents, antioxidant enzymes (SOD, CAT and APX) activity, and proline level in leaves was detected after using static MF (Rochalska, 2005; Hozayn and Qados 2010a; Hozayn and Qados 2010b; Dubois et al., 2013; Sadeghipour, 2015; Tehseen et al., 2016; Albys et al., 2018). The higher variation for both species observed in roots after treatment, could be associated with the mechanism that contributes to the osmotic adjustment since the root is a key organ involved in the uptake of water and nutrients, anchoring the plant to the substrate, and it is crucial for plant performance and crop productivity (Grossman and Rice, 2012; Mundim and Pringle, 2018). The great differences in sugars, amino acids, and polyols among organs, were previously reported in response to different stress conditions (Ullah et al., 2017; Borrelli et al., 2018; Skliros et al., 2018). The significant metabolite variation observed in durum wheat involved mainly sugar, while in lentil involved, other than sucrose, mainly organic acids, the stored pools of fixed carbon. This highlights the exclusivity of some compounds to the species studied and shows the first seedling development stage in both species can be significantly affected by the use of magnetized water and strictly related to morphological development. Selim et al. (2019) investigated the effect of irrigation with magnetized water in the leaves of two wheat cultivars under drought stress conditions, highlighting the best performances and significant increase of total soluble sugar concentration, total free amino acids, and proline after treatment. On the other hand, the results showed that the increase in the content of some metabolites is related to the use of magnetized water, as the consequence of the increased physical properties of water could affect the chemical and physical properties of polar metabolites. In general, the metabolites with osmoprotective functions significantly increased in almost all tissues and species analyzed, indicating a positive effect of using MWT, as MWT likely facilitates metabolite availability to act on their solubility. Among these metabolites, particular attention is reserved for threalose and mannitol in the root and epicotyl of durum wheat, and glycine and GABA in the seed and root of lentil, respectively. Moreover, Sadeghipour (2016) reported that magnetic water significantly improves WUE in some plants, especially those in regions where water resources are scarce, leading to an increase in plant development and crop productivity under reduced water input, which is of great importance for an arid environment suffering from frequent droughts (Selim and Selim, 2019; Liu et al., 2022). A deeper understanding of the effects of the MF on water and its influence on plant metabolism could revolutionize crop production under biotic and abiotic stress conditions, as well as water usage leading to crop yield improvement. However, since our study was done under controlled conditions, further investigations must be carried out that take into account the use of different genetic resources and must be repeated over time in open fields to evaluate the effect of the GE interaction. In addition to this preliminary study, other omics approaches could be used to gain a complete vision of the system biology and a deeper understanding of the metabolic mechanisms involved. A long-term cost/benefit study needs to be evaluated before the use of magnetized water can be considered a “green technology” accessible to all.

## Data availability statement

The original contributions presented in the study are included in the article/**Supplementary Material**. Further inquiries can be directed to the corresponding author.

## Author contributions

SS, CP conceived and designed the experiments. CP, DP, MAD collected the growth parameters. RB performed metabolomic analysis. SS, RB performed data statistical analysis, discussed the results, and wrote the manuscript. CP, DP carefully revised the manuscript. The authors read and approved the final manuscript.
