# Supplementary material for: Can the use of magnetized water affect the seedling development and the metabolite profiles of two different species: Lentil and durum wheat?
Source: Front Plant Sci. 2023 Feb 14;13:1066088. doi: 10.3389/fpls.2022.1066088 (PMC9971934; doi:10.3389/fpls.2022.1066088)
Supplement: Supplementary file 1 [file DataSheet_1.docx]

Supplementary Material

# Supplementary Figures and Tables

## Supplementary Figures


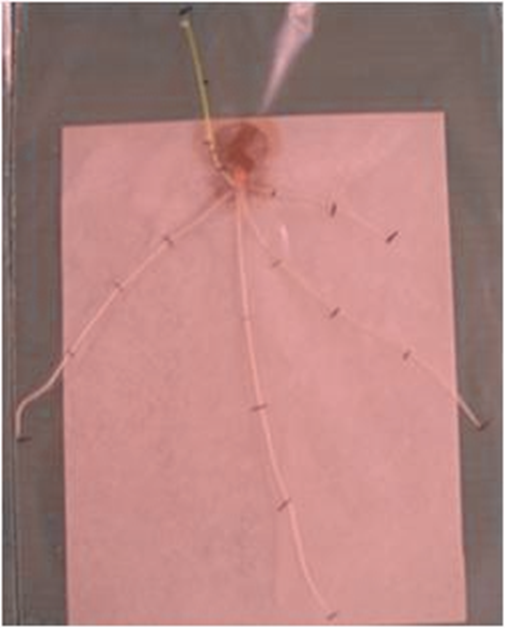

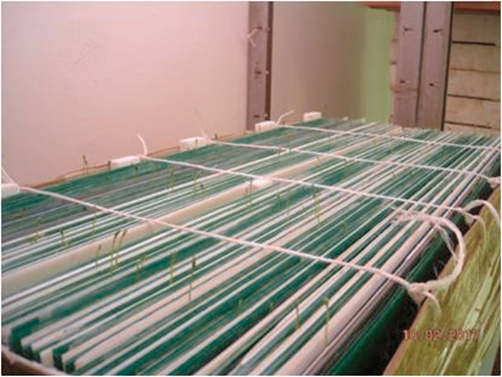


**Supplementary Figure 1.** Plant grown on paper positioned on non-transparent rigid plastic panels in the growth chamber


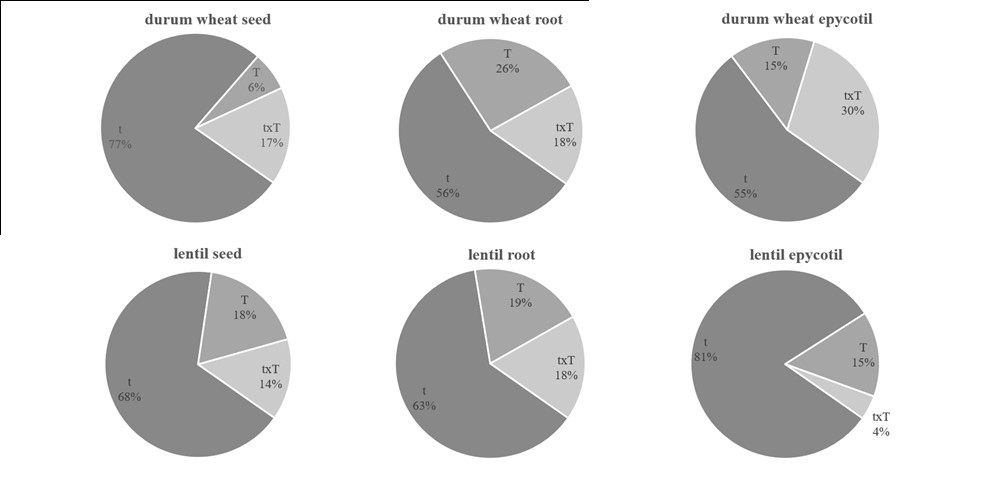


**Supplementary Figure 2.** Percentage of metabolite significant in seed, root and epicotyl of durum wheat and lentil.

**1.2 Supplementary Table**

**Supplementary Table 1.** Mean content (mg/Kg) of each metabolite detected in durum wheat and lentil seed at t0 time point.

| **Metabolite** | **Durum wheat** | **Lentil** |
| --- | --- | --- |
| valine | nd | 6.13 ± 1.19 |
| norvaline | 560.87 ± 0.58 | 5.48 ± 0.88 |
| proline | 454.03 ±0.90 | nd |
| serine | nd | 11.92 ± 0.72 |
| glutamic acid | nd | 28.65 ± 3.71 |
| **Amino acids** | **1014.90** | **52.17** |
| oxalic acid | 240.42± 35.35 | nd |
| phosphate | 20708.98 ± 717.09 | 182.77 ±44.95 |
| nicotinic acid | 15.59 ±0.08 | 318.95 ± 34.57 |
| maleic acid | 819.60 ± 17.16 | 13.37 ± 3.06 |
| succinic acid | 46.54 ± 3.88 | 187.25 ± 46.89 |
| fumaric acid | 211.36 ±9.39 | 1.76 ± 0.04 |
| malic acid | 333.15 ± 55.02 | 2192.19 ±622.24 |
| t-cinnamic acid | 13.38 ± 0.03 | nd |
| aconitic acid | nd | 2754.64 ± 136.12 |
| shikimic acid | 34.74 ± 0.23 | 226.25 ± 16.87 |
| citric acid | nd | 15207.02 ± 1522.10 |
| gluconic acid | nd | 11.33 ± 6.82 |
| quinic acid | 34.21 ± 0.04 | 28.89 ± 7.21 |
| **Organic acids** | **22457.98** | **21124.42** |
| xylose | 5.22 ± 0.19 | nd |
| lyxose | 2.48 ± 0.07 | nd |
| threalose | 320.05 ± 9.97 | nd |
| fructose | 665.64 ± 33.65 | 79.86 ± 18.81 |
| mannose | nd | 21.04 ± 0.79 |
| galactose | 794.45 ± 33.36 | nd |
| glucose | 388.93 ±18.47 | 150.09 ±34.56 |
| sucrose | 59202.40 ± 1048.70 | 24577.68 ± 4890.57 |
| lactulose | 3911.11 ± 137.15 | 1701.87 ± 315.66 |
| maltose | 9903.41 ± 417.64 | 3632.20 ± 908.12 |
| turanose | 23569.81 ± 1300.99 | nd |
| beta-gentibiose | nd | 94.05 ± 13.01 |
| isomaltose | nd | 23.55 ±2.13 |
| raffinose | 148521.13 ±10198.96 | 9562.59 ± 2244.01 |
| **sugars** | **247284.61** | **39842.93** |
| glycerol | 331.35 ± 11.92 | 15.36 ±2.47 |
| mannitol | 63.37 ±2.19 | 78.33 ± 19.84 |
| sorbitol | 41.15 ± 2.18 | 2.81 ±0.79 |
| myo-inositol | 256.60 ± 8.23 | 215.35 ± 51.03 |
| maltitol | nd | 23513.22 ± 5830.33 |
| **Sugar alcohols** | **692.46** | **23825.07** |
| decanoic acid | 269.95 ± 0.33 | nd |
| tetradecanoic acid | nd | 12.04 ± 0.11 |
| hexadecanoic acid | 19.35 ± 0.84 | 500.80 ± 12.28 |
| Octadecanoic acid | nd | 119.51 ± 0.59 |
| docosanoic acid | nd | 36.79 ± 0.14 |
| cis-9-hexadecenoic acid | 173.04 ± 0.01 | nd |
| cis,cis-9,12-octadecadienoic acid | 326.66 ± 1.65 | 113.40 ± 9.05 |
| cis-9-octadecenoic acid | 175.13 ±0.32 | 98.51 ±5.39 |
| cis,cis,cis-9,12,15-octadecatrienoic acid | 884.39 ± 0.31 | 133.92 ± 2.04 |
| cis-13-docosenoic acid | nd | 83.92 ± 0.22 |
| **Fatty acids** | **1848.51** | **1098.89** |
| octadecane | 242.30 ± 1.22 | nd |
| nonadecane | 272.13 ± 0.07 | nd |
| docosane | 83.70 ± 0.13 | nd |
| **Alkanes** | **598.13** | **nd** |
| 1-hexadecanol | 16.71 ± 0.02 | nd |
| 1-octacosanol | nd | 22.60 ± 0.20 |
| **Policosanols** | **16.71** | **22.60** |
| a-tocopherol | nd | 18.68 ± 0.43 |
| b-tocopherol | nd | 5.65 ± 0.17 |
| g-tocopherol | nd | 78.45 ±14.82 |
| **Tocopherols** | **nd** | **102.78** |
| 1,3-dihydroxy-5-nonadecylbenzene | 47.56 ±1.72 | nd |
| 1,3-dihydroxy-5-eicosylbenzene | 59.60 ± 0.17 | nd |
| **Alkylresorcinols** | **107.16** | **nd** |
| campsterol | nd | 451.29 ± 78.18 |
| b-sitosterol | nd | 176.14 ± 39.39 |
| stigmasterol | nd | 14.82 ± 2.69 |
| **Phytosterols** | **nd** | **642.25** |

**Supplementary Table 4**. Metabolites significant for T and t×T in durum wheat. Metabolites significant for both species in light grey; metabolites significant only in durum wheat in dark gray.

| **Metabolites** | **DW_Seed** | | **DW_Root** | | **DW_Epic** | |
| --- | --- | --- | --- | --- | --- | --- |
|  | **T** | **NT** | **T** | **NT** | **T** | **NT** |
| **isoleucine** | 327,53 ± 327,53 a | 235,95 ±235,95 a | 4285,32 ± 3422,07 a | 2261,29 ± 2262,29 b | 0,00 | 0,00 |
| **valine** | 102,53 ± 102,53 a | 77,17 ± 77,17 a | 399,25 ± 234,40 a | 73,37 ± 73,37 b | 0,00 | 0,00 |
| **succinic acid** | 0,00 | 0,00 | 134,80 ± 69,66 a | 40,02 ± 40,02 b | 0,00 | 0,00 |
| **fumaric acid** | 47,48 ± 23,88 a | 26,68 ± 26,68 b | 344,31 ± 49,63 a | 306,71 ± 37,45 a | 0,00 | 0,00 |
| **malic acid** | 140,92 ± 43,24 a | 146,26 ± 50,77 a | 10666,72 ± 3549,54 a | 7866,46 ± 3391,34 b | 141,51 ± 141,51 a | 233,80 ± 233,80 a |
| **oxalic acid** | 937,74 ± 783,53 b | 1704,55 ± 803,49 a | 1096,43 ± 103,46 a | 817,96 ± 207,72 a | 171,13 ± 171,13 a | 0,00 b |
| **phosphate** | 43864,03 ± 11396,83 a | 44959,51 ± 13591,83 a | 293845,31 ± 73688,44 a | 254177,71 ± 69517,33 b | 239031,93 ± 33556,88 a | 178381,71 ± 100030,36 b |
| **shikimic acid** | 20,20 ± 10,13 a | 20,39 ± 10,22 a | 128,59 ± 15,89 a | 114,14 ± 15,25 b | 125,49 ± 2,36 a | 138,39 ± 36,35 a |
| **galacturonic acid** | 396,76 ± 297,82 a | 474,48 ± 370,16 a | 2548,92 ± 911,65 a | 1861,99 b | 2410,10 ± 477,41 a | 2322,76 ± 1063,79 a |
| **mannitol** | 780,57 ± 495,43 a | 847,60 ± 533,48 a | 2767,84 ± 2025,06 a | 2337,87 ± 1881,01 a | 1071,07 ± 308,71 a | 690,23 ± 213,63 b |
| **sorbitol** | 185,27 ± 143,87 a | 253,90 ± 218,19 a | 110,38 ± 45,21 a | 79,96 ± 41,96 b | 137,33 ± 15,02 a | 75,00 ± 5,27 a |
| **myo-inositol** | 851,40 ± 374,58 a | 787,94 ± 323,64 a | 2054,57 ± 249,36 a | 1362,80 ± 282,92 b | 4257,56 ± 685,81 a | 3851,83 ± 2477,78 a |
| **xylose** | 56,98 ± 38,43 a | 51,03 ± 33,36 a | 90,71 ± 29,68 a | 71,17 ± 32,03 b | 0,00 | 0,00 |
| **lyxose** | 56,73 ± 40,08 a | 50,11 ± 34,19 a | 298,57 ± 153,57 a | 198,91 ± 135,71 b | 0,00 | 0,00 |
| **threalose** | 5035,49 ± 3789,50 a | 4542,78 ± 3281,57 a | 7283,30 ± 4030,54 a | 4897,16 ± 3626,96 b | 0,00 | 0,00 |
| **ribose** | 11,34 ± 10,46 a | 20,79 ± 19,19 a | 985,10 ± 514,29 a | 605,74 ± 434,27 b | 0,00 | 0,00 |
| **fructose** | 2887,52 ± 1740,26 a | 2984,22 ± 1932,07 a | 70006,03 ± 16767,85 a | 57548,31 ± 18170,22 b | 159320,16 ± 40078,73 a | 115253,69 ± 28918,10 b |
| **mannose** | 529,29 ± 406,19 a | 640,71 ± 481,85 a | 13649,97 ± 6936,41 a | 12617,73 ± 7170,35 a | 47681,44 ± 9787,81 b | 64535,37 ± 15681,41 a |
| **sucrose** | 90282,25 ± 13614,40 a | 89724,09 ± 14030,10 a | 148777,26 ± 38025,38 a | 113348,04 ± 15704,64 b | 170784,22 ± 32762,93 a | 200126,08 ± 13960,48 a |
| **lactulose** | 42804,50 ± 16617,91 a | 42124,97 ± 16858,22 a | 11720,07 ± 2451,10 a | 11652,40 ± 3287,70 a | 13604,19 ± 1198,20 a | 14820,15 ± 6473,86 a |
| **maltose** | 30881,50 ± 8708,31 a | 31243,38 ± 8556,18 a | 21233,29 ± 9417,08 a | 12551,00 ± 5236,86 b | 8350,79 ± 2744,35 a | 11749,11 ± 7235,67 a |
| **turanose** | 197617,51 ± 65752,32 a | 194604,18 ± 64957,77 a | 16128,50 ± 5224,41 a | 12523,26 ± 7291,07 b | 4731,65 ± 4731,65 b | 13384,23 ± 13384,23 a |
| **beta-gentibiose** | 3783,26 ± 2071,60 a | 3139,70 ± 1281,69 a | 3926,44 ± 2167,26 a | 2736,91 ± 1372,56 b | 4514,77 ± 443,88 a | 4793,04 ± 1196,92 a |
| **isomaltose** | 50981,21 ± 40457,56 a | 54862,40 ± 42713,15 a | 34997,81 ± 24562,84 a | 38923,75 ± 35623,85 a | 3116,36 ± 2507,32 a | 2792,81 ± 1760,91 a |
| **raffinose** | 7989,72 ± 3807,23 a | 5461,24 ± 1500,12 a | 0,00 | 0,00 | 0,00 | 0,00 |
| **decanoic acid** | 269,73 ± 0,20 a | 269,65 ± 0,11 a | 0,00 | 0,00 | 0,00 | 0,00 |
| **docosane** | 158,64 ± 62,14 a | 111,88 ± 17,93 b | 0,00 | 0,00 | 0,00 | 0,00 |
| **1,3-5-nonadecylbenzene** | 42,65 ± 3,98 a | 40,89 ± 2,90 a | 0,00 | 0,00 | 0,00 | 0,00 |
| **1,3-5-eicosylbenzene** | 58,56 ± 0,16 b | 60,63 ± 1,81 a | 0,00 | 0,00 | 0,00 | 0,00 |
| **b-sitosterol** | 0,00 | 0,00 | 7227,65 ± 2735,69 a | 5453,60 ± 1809,51 b | 3024,52 ± 1927,57 a | 4034,26 ± 3671,10 a |

The metabolite not significant for T are significant for txT interaction

**Supplementary Table 5.** Metabolites significant for t and t×T in lentil. Metabolites significant for both species in light grey; metabolites significant only in lentil in gray.

| **Metabolites** | **L_Seed** | | **L_Root** | | **L_Epic** | |
| --- | --- | --- | --- | --- | --- | --- |
|  | **T** | **NT** | **T** | **NT** | **T** | **NT** |
| **glycine** | 2891,66 ± 1557,42 a | 2685,27 ± 1388,41 b | 0,00 | 0,00 | 531,29 ± 169,00 a | 438,94 ± 119,93 a |
| **glutamic acid** | 75,36 ± 22,84 a | 30,71 ± 12,72 b | 732,00 ±688,67 a | 464,47 ± 454,77 b | 41,86 ± 19,73 b | 88,74 ± 10,15 a |
| **phenyl alanine** | 0,00 | 0,00 | 6,52 ± 6,52 a | 2,87 ± 2,87 b | 0,00 | 0,00 |
| **serine** | 225,75 ± 44,99 a | 220,19 ± 58,10 a | 195,30 ± 175,59 a | 159,97 ± 151,78 b | 158,14 ± 0,32 b | 250,44 ± 10,04 a |
| **threonine** | 501,83 ± 222,91 a | 459,25 ± 137,47 a | 651,45 ± 582,08 a | 449,21 ± 437,64 b | 331,16 ± 111,60 b | 491,90 ± 148,73 a |
| **valine** | 653,15 ± 441,93 a | 461,92 ± 259,41 b | 1486,10 ± 1269,38 a | 1016,42 ± 978,71 b | 1220,94 ± 265,81 a | 1266,77 ± 173,77 a |
| **GABA** | 23,32 ± 23,32 a | 12,53 ± 12,53 a | 51,22 ± 51,22 a | 37,63 ± 37,63 b | 0,00 | 0,00 |
| **cadaverine** | 1,94 ± 1,00 a | 2,03 ± 1,08 a | 246,64 ± 223,45 a | 160,07 ± 139,41 b | 62,53 ± 16,00 a | 74,85 ± 14,50 a |
| **aconitic acid** | 3482,83 ± 754,79 a | 3188,82 ± 602,09 a | 7835,88 ± 4594,14 a | 8147,62 ± 4211,24 a | 8786,90 ± 1470,16 a | 8647,97 ± 1058,88 a |
| **isocitric acid** | 0,00 | 0,00 | 527,69 ± 527,69 a | 186,96 ± 186,96 b | 0,00 | 0,00 |
| **succinic acid** | 159,17 ± 30,37 a | 111,79 ± 16,56 b | 300,08 ± 300,08 a | 268,40 ± 268,40 a | 836,02 ± 233,27 a | 875,03 ± 281,35 a |
| **malic acid** | 1296,06 ± 93,10 a | 1354,75 ± 81,22 a | 18829,91 ± 17140,47 a | 14165,57 ± 13028,06 b | 15567,06 ± 2007,60 a | 15278,97 ± 1978,12 a |
| **phosphate** | 1282,09 ± 458,22 a | 1232,12 ± 396,44 a | 2265,16 ± 1211,07 a | 1990,94 ± 1243,95 b | 2493,71 ± 267,25 a | 2343,12 ± 159,80 a |
| **gluconic acid** | 7,63 ± 7,63 a | 8,02 ± 8,02 a | 4317,13 ± 4194,73 a | 774,14 ± 685,84 b | 6315,44 ± 5760,43 a | 999,91 ± 430,66 a |
| **mannitol** | 67,18 ± 23,07 a | 46,21 ± 5,87 a | 584,51 ± 346,67 a | 1183,49 ± 963,50 a | 941,51 ± 735,80 a | 169,11 ± 13,52 b |
| **sorbitol** | 33,89 ± 1,95 a | 33,11 ± 2,81 a | 320,74 ± 151,35 a | 286,60 ± 145,24 a | 592,33 ± 27,03 a | 550,76 ± 35,21 a |
| **myo-inositol** | 1647,84 ± 394,28 a | 1668,62 ± 320,19 a | 1276,97 ± 437,76 a | 1130,57 ± 453,31 a | 965,56 ± 335,63 a | 845,12 ± 370,08 b |
| **maltitol** | 12452,78 ± 4461,53 b | 14998,82 ± 5811,11 a | 8895,36 ± 3941,23 a | 9173,38 ± 5120,89 a | 3153,18 ± 1002,67 a | 2543,94 ± 144,82 b |
| **xylose** | 24,19 ± 8,08 a | 19,36 ± 5,33 b | 109,06 ± 109,06 a | 125,95 ± 125,94 a | 174,85 ± 49,39 a | 172,75 ± 39,53 a |
| **ribose** | 13,94 ± 7,97 a | 6,17 ± 4,06 b | 347,59 ± 62,93 a | 307,21 ± 28,92 a | 217,28 ± 22,81 a | 198,44 ± 9,93 b |
| **galactose** | 51,21 ± 10,07 a | 44,61 ± 7,83 a | 98,76 ± 66,93 a | 51,13 ± 25,78 a | 44,01 ± 9,15 a | 35,85 ± 13,94 a |
| **sucrose** | 77188,77 ± 21973,47 b | 84722,80 ± 21039,64 a | 77768,84 ± 3577,72 a | 70933,54 ± 11622,35 a | 47688,05 ± 5791,79 a | 45932,48 ± 9344,01 a |
| **maltose** | 17672,83 ± 5799,38 b | 20158,02 ± 6933,97 a | 6925,26 ± 3293,14 a | 7131,64 ± 3609,70 a | 3702,07 ± 338,27 a | 3946,63 ± 327,33 a |
| **beta-gentibiose** | 482,74 ± 133,56 b | 529,89 ± 157,97 a | 506,95 ± 280,54 a | 437,19 ± 218,59 a | 148,90 ± 43,13 a | 114,66 ± 20,30 a |
| **tetradecanoic acid** | 9,82 ± 4,91 a | 9,88 ± 4,94 a | 43,16 ± 1,53 a | 41,69 ± 0,90 b | 38,71 ± 0,73 a | 39,43 ± 1,16 a |
| **hexadecanoic acid** | 150,31 ± 16,49 b | 348,20 ± 194,20 a | 417,57 ± 37,00 a | 400,10 ± 9,40 a | 362,68 ± 93,66 a | 354,17 ± 87,19 a |
| **g-tocopherol** | 158,55 ± 9,11 a | 115,95 ± 58,52 b | 23,23 ± 23,23 a | 20,01 ± 20,01 b | 25,72 ± 25,72 a | 25,78 ± 25,78 a |
| **campsterol** | 818,69 ± 299,73 a | 786,31 ± 228,26 a | 1899,84 ± 357,41 a | 1703,86 ± 287,90 a | 1252,83 ± 482,05 a | 1357,95 ± 608,82 a |
| **fucosterol** | 7,79 ± 7,79 a | 0,00 b | 87,25 ± 13,72 a | 83,19 ± 10,10 a | 43,39 ± 43,39 a | 45,37 ± 45,37 a |

The metabolite not significant for T are significant for txT interaction
